# Effectiveness of thinner polyvinyl alcohol fibers on mechanical properties and cost effectiveness of office automation floor panels

**DOI:** 10.1371/journal.pone.0324126

**Published:** 2025-05-22

**Authors:** Tek Raj Gyawali

**Affiliations:** School of Engineering, Pokhara University, Pokhara, Nepal; Ural Federal University named after the first President of Russia B N Yeltsin Institute of Physics and Technology: Ural'skij federal'nyj universitet imeni pervogo Prezidenta Rossii B N El'cina Fiziko-tehnologiceskij institut, RUSSIAN FEDERATION

## Abstract

Floor finishing is the final and crucial stage in building construction. Flooring materials must possess strength, insulation, durability, aesthetics, and comfort. Office buildings typically use raised floor systems, known as office automation (OA) floors, which utilize various panel types for insulation and wiring purposes. Polyvinyl alcohol (PVA) fiber-reinforced mortar, with a water-cement (W/C) ratio of 0.30, a cement-sand (C:S) ratio of 1:0.776, and a fiber content of 3.0% by volume (fiber size: Ф100µm × 12mm), is mechanically pressed with water dehydration to create OA floor panels measuring 500 mm in length and width and 23 mm in thickness. A W/C ratio of 0.32 was maintained to achieve the same workability for producing panels using thinner PVA fibers (fiber size: Ф40µm × 12mm). A comparative analysis of both panel types was conducted, focusing on mechanical properties and costs. Panels reinforced with thinner PVA fibers exhibited superior performance in resisting compressive and impact loads. This enabled a reduction in fiber content to 1.2% (a 60% decrease) and panel thickness to 22 mm (a 4.35% decrease) compared to panels with thicker fibers. Consequently, the cost of panels with thinner fibers was reduced by 43.9%. The findings demonstrate that thinner PVA fibers are a viable replacement for thicker fibers in conventional OA floor panels, significantly reducing costs while meeting the required mechanical properties. The implementation of these findings in real OA floor factories reduces the consumption of PVA fibers and cement, contributing to sustainable development by lowering the carbon footprint, which is primarily generated during the production of cement and PVA fibers.

## Introduction

Floor finishing is the final and one of the most important stages in building construction before handing it over to the client for use [1]. The flooring relates to the floor surface’s regularity in terms of levelness and flatness. Levelness refers to longer distances of more than 3 m, and flatness refers to shorter distances of about 300 mm [2,3]. Lime-based plastering is believed to have started around 7500 BC in Jordan. The history of flooring began around 3000 BC, when the Egyptians used bricks and stones for flooring. The Greeks created pebble mosaics in 1000 BC. This was followed by wooden flooring (400 AD), rubber flooring (1200 AD), rug flooring (1502 AD), carpet flooring (1791 AD), and polyvinyl chloride or vinyl flooring (1926 AD) [4]. In reinforced concrete buildings, the conventional method of flooring involves screeding with lean cement mortar, followed by plastering with cement paste, and then carpeting [5].

Currently, different types of flooring materials are used in building construction, and their selection depends on the application, aesthetics, and the user’s preference [6]. Flooring materials not only provide aesthetic value and comfort in buildings but also offer acoustic insulation and water resistance [1]. These materials should not only cover the floor but also provide the required quality at a moderate cost [7]. Engineering design is necessary for selecting such materials, helping decision-makers choose the most appropriate options [8]. Many international standards have been developed for the selection and application of these materials, including various tests and code provisions. The selection criteria for materials require a broader scope of evaluation, incorporating different tests and code provisions [8,9]. Valuable engineering is developed to analyze the selection of materials based on consistent performance, reliability, and maintainability at the lowest cost [10].

The type of flooring generally varies depending on the type of building, such as residential, commercial, office, hospitals, etc. This article focuses on the flooring of office buildings. Office flooring began in the early 1700s with the use of linoleum. Asphalt and vinyl flooring replaced linoleum by the 1930s. Rubber flooring became commercially available in the mid-19th century and was widely used by the early 20th century [11]. In the 21st century, office automation (OA) flooring is becoming popular. OA flooring is a raised floor, also called a free access floor, with panels arranged 30–100 mm above the original floor. The gap between the two floors is used for setting electrical wiring, LAN cables, power cords, telephone wiring, etc [12].

OA floor panels are generally placed on the floor, simply supported at four corners. Typically, the surface of the panel is square, with each side ranging from 500 to 600 mm [13]. The thickness of the panels depends on their load-resisting capacity. The required load-resisting capacity depends on the functionality of the office and may range from 3000 N to 5000 N [14]. JIS A 1408 [15] recommends that each OA floor panel should resist a compressive load of 3922.7 N, with deflection within 5.0 mm. It further recommends that the panel should resist the designed impact load without forming any cracks, and the residual deflection should be within 3.0 mm.

The use of polyvinyl alcohol (PVA) fiber in mortar and concrete began after the invention of PVA fiber in Japan in 1950 [16]. PVA fiber has high tensile strength and is lightweight. It is generally used in cement mortar composites [17]. PVA fiber-reinforced mortar composites are utilized in building walls, permanent formworks, bridge deck slabs, flooring panels, windproof panels for railways, retrofitting, shotcrete for slope stabilization and tunnel linings, overlays for concrete road/airport pavement, etc. [16]. Engineering cementitious composites were developed using PVA fibers to increase the flexural strength and ductility of cement mortar [17–19]. PVA fiber also enhances fracture toughness and crack resistance [20]. The autogenous and dry shrinkage of mortar composites can also be reduced by 30% with the use of PVA fiber [20]. Most previous researchers have concentrated on the flexural strength [21–23], bridging effect [24], crack control [25,26], toughness [27,28], flexural performance [29], and fracture energy [30] of PVA fiber composites. However, no research article has been found on OA floor panels using PVA fiber.

Different sizes of PVA fiber, varying in diameter and length, are commercially available in the market. The thicker RECS100 PVA fiber (Ф100 mm × 12 mm (diameter × length)) can be uniformly distributed inside the cement mortar using conventional mixing methods [31]. However, with highly workable mortar, the coating layer of the fiber was found to be thinner around the glassy surface of the fiber. The High Ductile Mortar (HDM) mixing method was developed to uniformly disperse and firmly coat thin PVA fibers inside mortar composites [32,33].

The commercial production of OA floor panels by Fujimi Koken in Japan is based on a methodology where workable PVA mortar, using the RECS100 fiber, is first produced. This mortar is then pressed into the form of panel sizes, dehydrating the excess water, and demoulded for autoclaved curing [34]. However, the use of the HDM mixing method was not possible for producing OA panels using thinner (Ф40 mm × 12 mm) (REC15) fibers. This paper discusses the use of a modified HDM mixing method to compare the required properties of OA floor panels using the thinner REC15 PVA fibers with those using thicker RECS100 PVA fibers. The hypothesis is that if the modified HDM mixing method provides better performance, the content of REC15 fiber as well as the thickness of the panels can be decreased, ultimately reducing the cost of OA floor panels. It not only reduces the cost of the panels but also contributes to the sustainability of OA floor panels by lowering the carbon footprint, which is typically generated during the production of cement and PVA fibers.

## Materials and methods

### Materials’ properties

The type of cement used was high early-strength Portland cement (Type III). Its specific gravity and surface area (Blaine value) were measured according to JIS R 5201 [35], with values of 3.15 and 449 m²/kg, respectively. The major compounds, measured according to JIS R 5204 [36], are listed in Table 1.

**Table 1:** Compounds of Type III Portland cement.

Types	Content (%, by weight)
**Lime (CaO)**	65.2
**Silica (SiO**_**2**_)	21.0
**Alumina (Al**_**2**_**O**_**3**_)	5.28
**Iron Oxide (Fe**_**2**_**O**_**3**_)	2.56
**Sulfur trioxide (SO**_**3**_)	2.10
**Tri-calcium Silicate (C**_**3**_ **S)**	60.4
**Di-calcium Silicate (C**_**2**_ **S)**	14.7
**Tri-calcium Aluminate (C**_**3**_ **A)**	9.66
**Tetra-calcium aluminoferrite (C**_**4**_ **AF)**	7.79
**Others**	3.88

The required properties of the sand were tested before the experiments and are provided in Table 2.

**Table 2:** Required properties of sand.

Properties	Values
**Type**	Crushed
**Maximum size**	5.0 mm
**Specific gravity**	2.66
**Fineness modulus**	2.38
**Water absorption**	1.39%

PVA fiber is lightweight and has high tenacity, high modulus, low elongation, good adhesion to the cement matrix, and better resistance to chemicals. The type of PVA fiber used in the conventional production method is RECS100, while REC15 type PVA fiber is used in the HDM mixing method. The use of PVA fibers in mortar/concrete improves ductility, tensile and flexural strengths, and crack-controlling capacity (development of multiple cracks before failure). REC15 type PVA fiber is used in permanent formwork to enhance toughness and crack control [37]. Table 3 shows the basic characteristics of both types of PVA fiber.

**Table 3:** Basic characteristics of REC15-type and RECS100-type PVA fibers [37].

Parameters	REC15	RECS100
**Diameter (mm)**	0.04	0.1
**Length (mm)**	12	12
**Specific gravity**	1.3	1.3
**Tensile strength (MPa)**	1600	1200
**Young’s modulus (GPa)**	41	28
**Fiber Elongation (%)**	6	6

From the table, it is evident that the diameter of REC15 type fiber is 40% less than that of the RECS100 type fiber. The tensile strength and Young’s modulus of REC15 fiber are 33.3% and 46.4% higher, respectively, than those of the RECS100 type fiber. The water used was potable with a pH value of 7.0.

### Conventional mixture proportions and amendment for HDM mixing method

In the HDM mixing method, superplasticizer and viscosity agent are added to enhance the viscosity and workability of the mortar. The viscosity agent (powder) is premixed with cement, while the superplasticizer (liquid) is mixed with the water. The viscous mortar is prepared by mixing cement, sand, and the first portion of water. Next, PVA fibers are uniformly dispersed and coated with the thick viscous mortar during mixing. Finally, the workability of the PVA mortar is increased by adding the second portion of water without compromising the coating condition of the fibers [37].

In the trial tests, the same conventional mixture proportion of the base mortar (Table 4) was used for the HDM mixing method, with the addition of superplasticizer and viscosity agent. The PVA mortar was prepared using 1.2% thinner REC15 PVA fiber. The PVA mortar exhibited good dispersion and coating of the thin PVA fiber, as well as good workability. However, during pressing to form the shape of the OA floor panel, this PVA mortar failed. The water could not be sufficiently dehydrated, and the surface did not achieve a smooth finish due to mortar sticking to the upper pressing plate, attributed to its high viscosity. Moreover, the panel collapsed during demolding. The PVA mortar (with REC15 fiber) failed even without the use of superplasticizer and viscosity agent due to insufficient workability, caused by the increased total surface area to be coated by the thinner fiber.

**Table 4:** Conventional and amended HDM mixture proportions of PVA mortar.

Mixing method	Conventional method	HDM method
**Fiber type**	RECS100	REC15
**W/C ratio**	0.30	0.32
**Water (kg/m**^**3**^)	320	340
**Cement (kg/m**^**3**^)	1067	1067
**Sand (kg/m**^**3**^)	828	855
**Fiber (kg/m**^**3**^)	39	ranging
**Naming**	RECS100 CM	REC15HDM

In the conventional mixture proportion using thicker RECS100 fiber), the PVA mortar had a water-cement ratio of 0.3, with 320 kg/m³ of water, 828 kg/m³ of sand, and a fiber content of 3.0% by total volume (39 kg/m³). During pressing to produce the OA floor panel, a certain amount of excess water was dehydrated. Considering this issue, adjustments were made to address the mixing problem of PVA mortar with thinner REC15 fiber using the modified HDM mixing method for OA floor panel production.

The basic conventional mixture proportion was maintained, increasing the water-cement ratio from 0.30 to 0.32, while keeping the cement content the same (1067 kg/m³) as in the conventional mixture. This adjustment increased the unit content of water by 20 kg/m³ compared to the conventional mixture, providing a similar level of workability for pressing the PVA mortar to form the OA floor panel. The fiber content was adjusted accordingly, and the sand content was also modified.

For mixing PVA mortar using REC15 fiber, a modified version of the HDM mixing method was adopted. The water was divided into two parts, with the first part being the same amount as in the conventional mixture proportions. The mortar using the first part of the water was prepared, and REC15 fibers were mixed with it to ensure uniform distribution and firm coating of mortar around each fiber. Then, the second part of the water was added and mixed to increase the workability of the PVA mortar without disturbing the coating condition of the fibers. After pressing to form the designed panel, the amount of water remaining in the panel was confirmed to be the same for both types of panels, maintaining a W/C ratio of 0.24.

Table 4 provides the mixture proportions for the conventional PVA mortar and the amended HDM mortar. The mixture proportion using RECS100 PVA fiber and mixed with the conventional method was named “RECS100CM”, while that using REC15 PVA fiber and mixed with the HDM mixing method was named “REC15HDM”.

### Mixing procedure and production method

The Omni type mixer (manufactured in Japan), with a capacity of 100 liters, was used for mixing the PVA mortar. It is a revolutionary diffusion mixing device that does not use stirring blades. Instead, it is equipped with flexible rubber balls attached to a vibrating plate. The mixer accelerates the mixture and shakes it in random directions, allowing for rapid mixing in a short amount of time.

**Fig 1:**
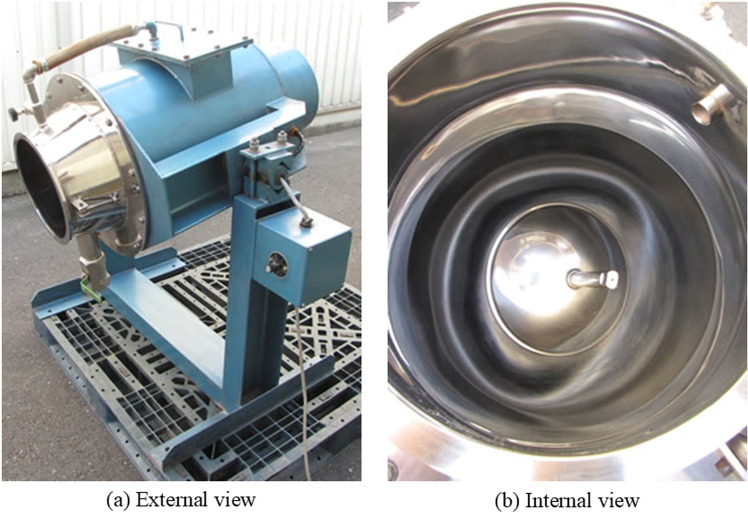
External and internal view of the Omni mixer [ 38].

The standard OM Mixer uses this innovative mechanism without stirring blades, making it suitable for mixing various materials with drastically different specific gravities, fragile raw materials, metal powder materials, as well as high-viscosity resins such as epoxy, mold release agents, and additives. It is also effective for dispersing and mixing fiber materials like GRC (alkali-resistant glass fiber reinforced cement), peat moss, and other fibrous substances. The mixer performs soft mixing without causing damage to the mixture (such as particle breakage or fiber bending), ensuring excellent performance. The external and internal view of the Omni mixer is shown in Fig 1 [38].

In the conventional mixing method, all ingredients of the base mortar were automatically charged and mixed for 3 minutes. Then, PVA fiber was automatically charged and mixed for another 2 minutes to prepare the PVA mortar. In the modified HDM mixing method, the sand, cement, and the first part of the water were charged and mixed for 2 minutes to prepare the viscous mortar. Then, PVA fiber was charged and mixed for another 2 minutes. Finally, the second part of the water was charged and mixed for 1 minute to increase workability. The total mixing time was kept the same (5 minutes) for both mixing methods.

After mixing the PVA mortar, it was poured into the hopper positioned above the plate of the OA floor production system. The required volume of PVA mortar was automatically supplied to the bottom plate, adjusting the 500mm × 500mm mold whose depth could be varied for producing panels of different thicknesses. Subsequently, the PVA mortar was pressed by the upper plate to maintain the required size and thickness of the OA floor panel, facilitating the dehydration of excess water.

**Fig 2:**
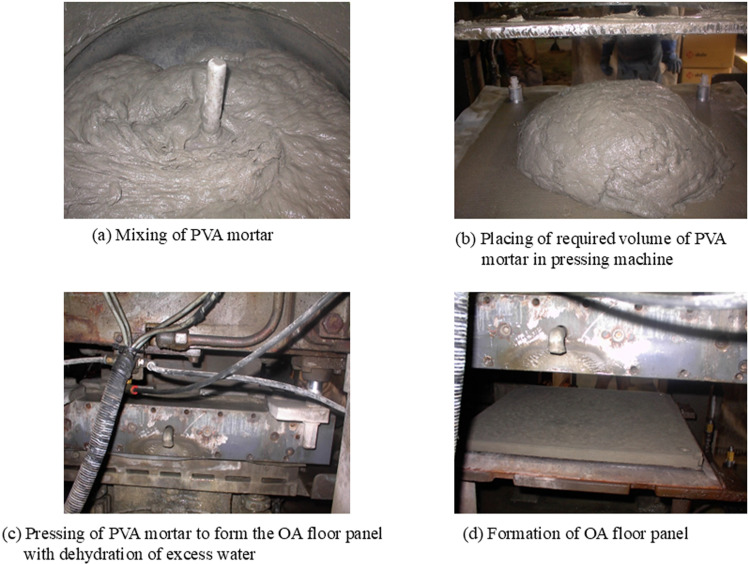
Mixing of PVA mortar and production method of OA floor panel.

The mixing and production flow of the OA floor panel is illustrated in Fig 2. For the conventional production method, only one type of panel with 3% RECS100 fiber content and a thickness of 23mm was produced. In this study, using the modified HDM mixing method, panels of various thicknesses and fiber contents were produced to facilitate comparison with the conventional panel.

After casting the panels, they were transferred to undergo the autoclave curing procedure. The strength tests of the panels were conducted at the age of 28 days.

### Strength tests of OA floor panels

**Fig 3:**
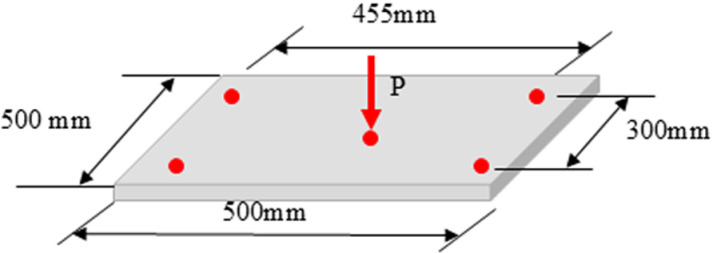
Adjustment of the OA floor panel for both static and impact loading.

The JIS A 1408 [15] method was used for both static and impact loading tests. Fig 3 illustrates the setup conditions of the panel and static loading applied to it. The panel was supported at four corners for both types of loading, maintaining effective spans of 455 mm and 300 mm on the longitudinal and transverse sides, respectively. In the figure, “P” indicates the monotonic load for static monotonic loading and the impact load for impact loading.

**Fig 4:**
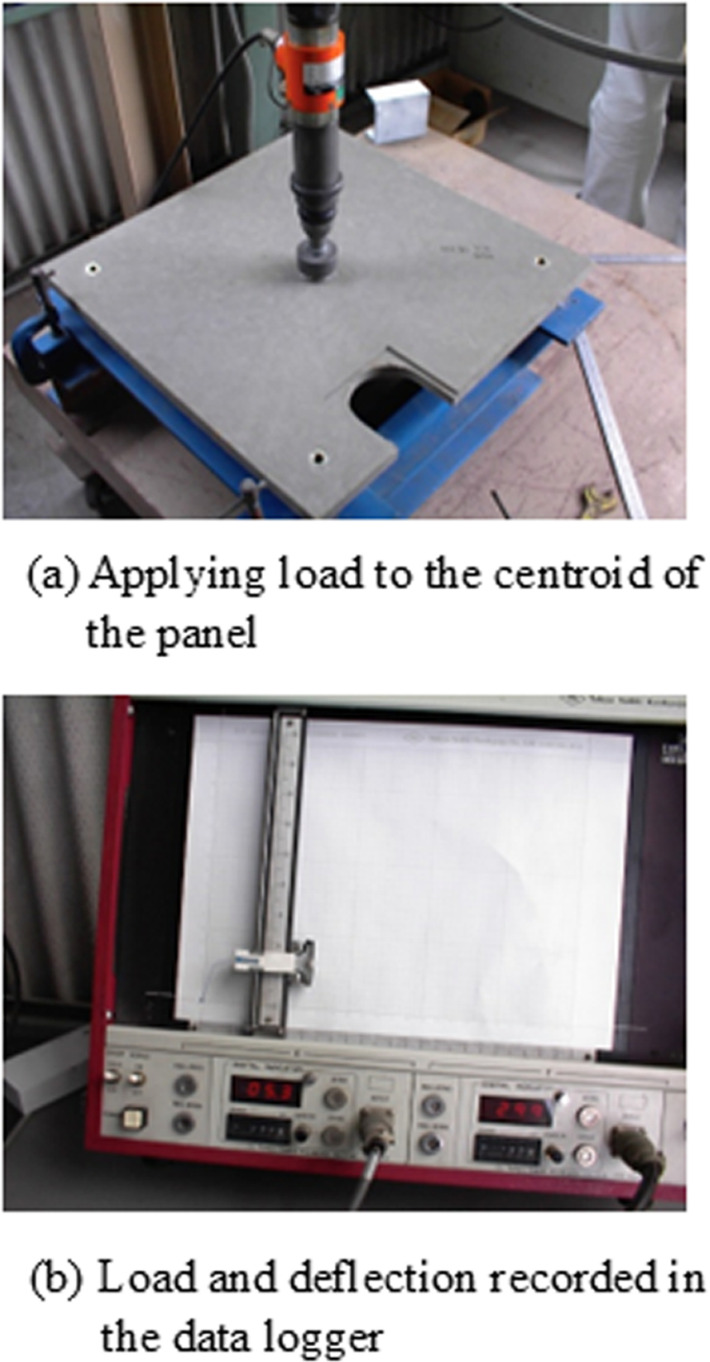
Static loading procedure.

For static loading, the load was applied monotonically to the centroid of the panel at a rate of 14 MPa/minute until the panel failed. The applied load and deflection were recorded using a data logger. A load-deflection curve was also plotted for visual inspection. The testing procedure for static loading is depicted in Fig 4. Three panels were produced and tested under different parameters.

From the static loading test, average load-deflection curves were generated and analyzed to determine the necessary panel thickness with the minimum fiber content required.

**Fig 5:**
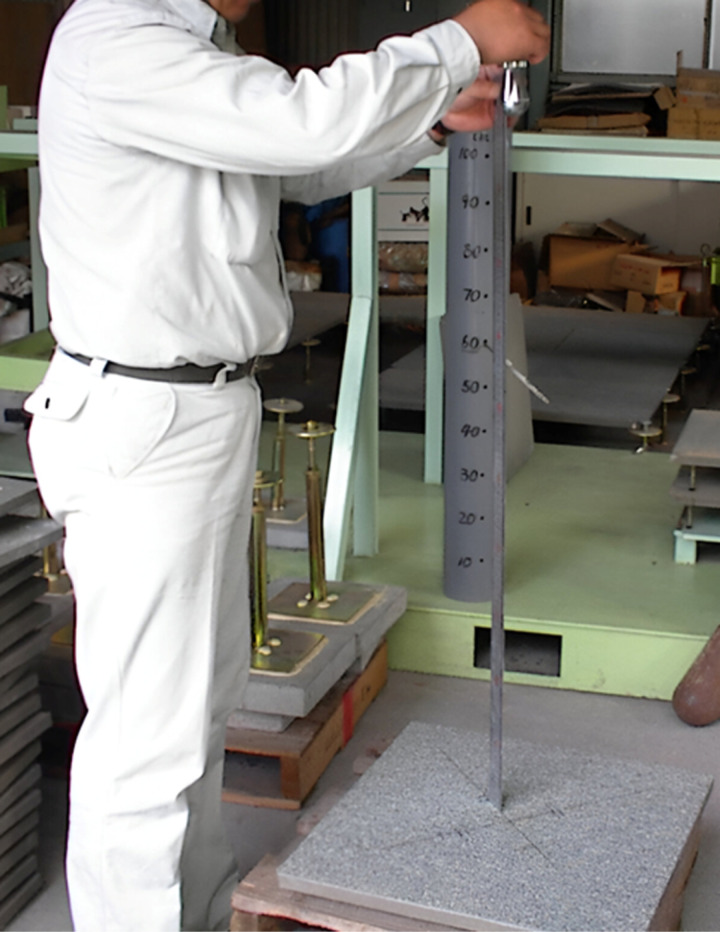
Impact test procedure on panel.

The procedure for the impact test is illustrated in Fig 5. As depicted, a 500-gm eggplant-shaped steel ball with a diameter of 42 mm was dropped from a height of 1 m. Following the ball drop test, inspections were conducted to detect any cracks, measure the maximum deflection, and assess the residual deflection.

Please note that three panels were used in each respective test, and average values were calculated for further direct and indirect analysis. It was confirmed that the standard deviation of the three respective datasets was within the margin of error.

## Results and discussions

### Visual observation, workability, and produced floor panel

**Fig 6:**
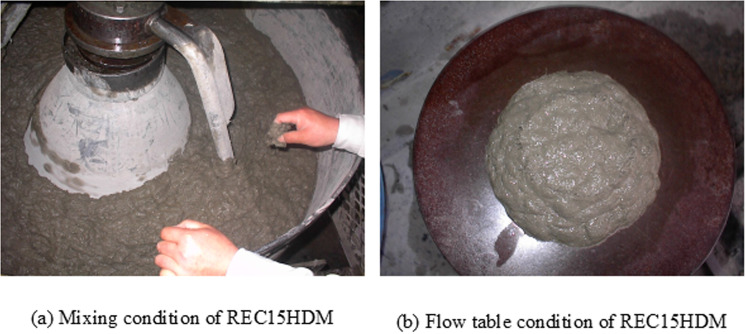
Mixing and flow condition of REC15HDM.

The modified mixture proportions of the PVA mortar allowed the HDM mixing method to produce panels with thinner PVA fibers. The mixing condition of REC15HDM was similar to that of RECS100 CM. The mortar had good workability, with a flow table value of more than 250 mm. The PVA fibers were uniformly distributed and firmly coated throughout the mortar, as confirmed visually and by hand touch. Fig 6 illustrates the mixing and flow table condition of REC15HDM.

**Fig 7:**
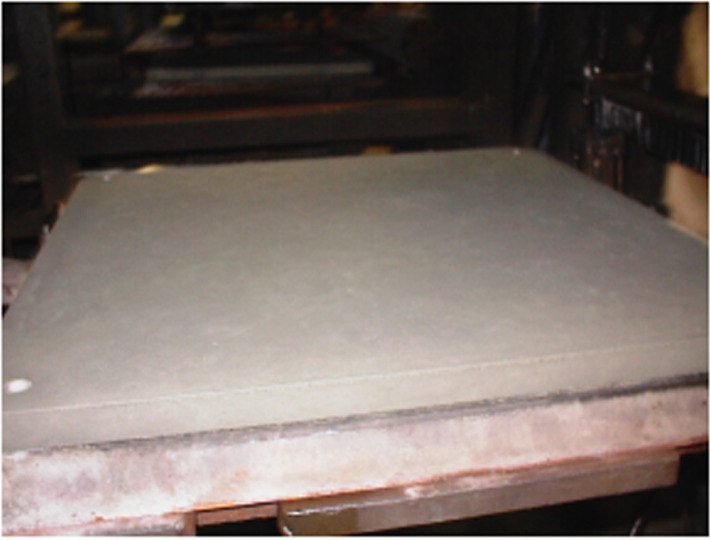
Visual condition of REC15HDM panel after production.

All the panels produced for the tests had a smooth surface with accurate length, breadth, and thickness. No voids or uneven distribution of non-coated fibers were observed on the surface of the panels. The surface of the REC15HDM was smoother than that of RECS100 CM due to the thinner size of the PVA fibers. Fig 7 illustrates the condition of the REC15HDM panel just after production.

### Central point loading

**Fig 8:**
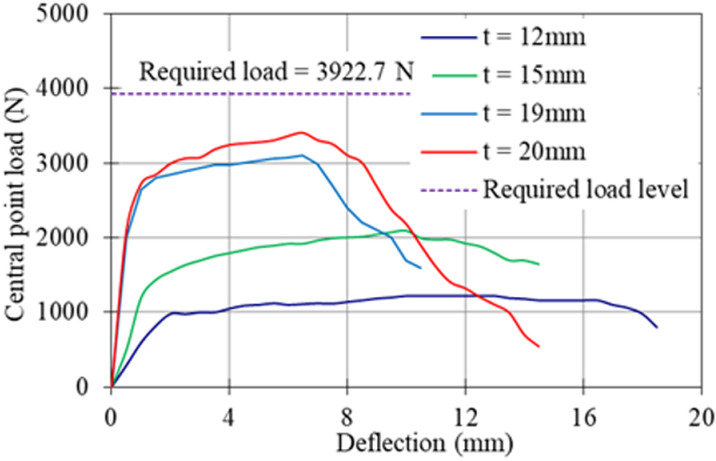
Central point load-deflection curves of the panel with different thickness.

Fig 8 shows the average load-deflection curve for central point loading of the panels with varying thicknesses. The panel with 12 mm thickness displayed a flat load-deflection curve after the yield point, indicating that deflection continued to increase while the load remained constant. The deflection-hardening effect (i.e., the increase in load-resisting capacity with increased deflection after the yield point) was negligible.

The deflection-hardening effect refers to an increase in load-resisting capacity with an increase in deflection after the yield level. Conversely, if the load-resisting capacity decreases with increased deflection, it is called the deflection-softening effect. These phenomena are commonly referred to as strain-hardening or strain-softening effects in the context of stress-strain curves. A flat curve indicates no sign of either strain-hardening or strain-softening effect.

The panel with 15 mm thickness showed more load-hardening than the 12 mm thick panel. The deflection-hardening effect was significantly increased in the 19 mm and 20 mm panels. From these test results, it is easily understood that increasing the thickness of the panel increases the load-resisting capacity.

**Fig 9:**
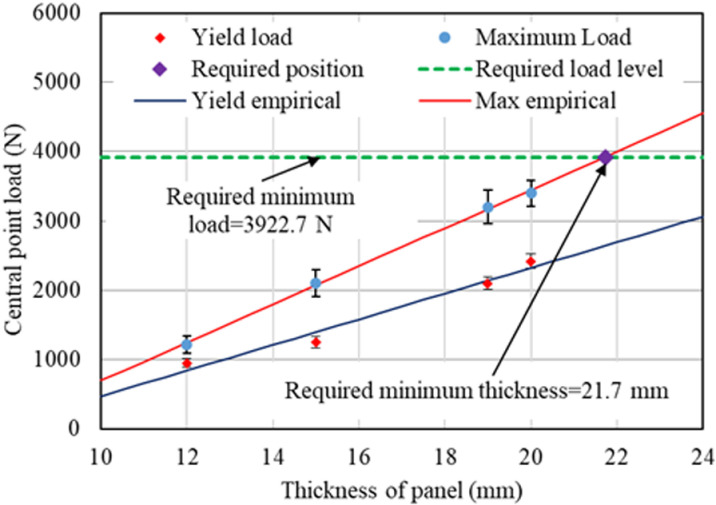
Relation of yield load and maximum load with thickness of panel.


From the load-deflection curve of the test results for each panel, the yield load and maximum load were identified, and the average values were plotted on a graph with varying panel thicknesses. Fig 9 shows the relationship between yield load and maximum load with panel thickness. The graph indicates that both the yield load and maximum load increased linearly with the increasing thickness of the panel. The R² value of the linear regression was 0.970 for the yield load and 0.999 for the maximum load. To represent these experimental results, an empirical model for both yield and maximum loads was developed, as shown in Equation (1).

$$P_{c}=at-b$$ (1)

Here, $P_{c}$ is the central point load and t is the thickness of the panel. a and b are empirical constants. The values of a and b were 185 and 1377, respectively, for the yield load, and 275 and 2052, respectively, for the maximum load. The greater value of b for the maximum load compared to the yield load indicates that the fiber-reinforced mortar/concrete consistently exhibits ductility behavior with a strain-hardening (deflection-hardening in this study) effect, resulting in the maximum load being greater than the yield load. The greater value of a for the maximum load compared to the yield load obtained in this study suggests that the increase rate of the maximum load is relatively higher than that of the yield load as the panel thickness increases.

JIS A 1408 [16] recommends that the panel should resist a minimum load of 3922.7 N, with a deflection within 5.0 mm. From these test results, even the 20 mm REC15HDM panel could not meet both requirements. The maximum load it resisted was only 3400 N, which was 13.3% less than the required load. The deflection at this load was recorded as 6.5 mm, which was 30% more than the allowable deflection. Analysis of the test data indicates that the panel should have a minimum thickness of at least 21.7 mm to meet the load target, with a maximum deflection not exceeding 5 mm. This investigation revealed that the thickness of REC15HDM should not be reduced below 22 mm.

**Fig 10:**
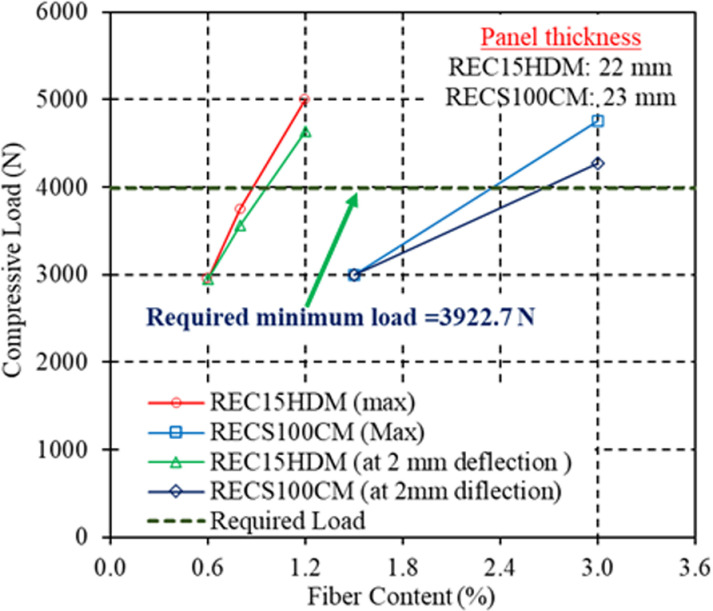
Effect of varying content of PVA fibers on load resistivity capacity of OA floor panels.

Thus, 22 mm thick REC15HDM panels and 23 mm thick RECS100 CM panels were produced with varying contents of REC15 (0.6%, 0.8%, and 1.2%) and RECS100 (1.5% and 3.0%), respectively, and their load resistivity capacities were tested and compared. The comparison results are shown in Fig 10. With 0.6% REC15 content, the maximum compressive load was 2950 N. The load capacity increased to 3750 N (a 27.1% increase) when the fiber content was increased from 0.6% to 0.8%. However, the load capacity was still lower than the required load of 3922.7 N. When the fiber content was increased to 1.2%, the load capacity rose to 5002 N (a 69.6% increase), which was significantly higher than the required load capacity. In the case of RECS100 CM, the load capacities were 3000 N and 4758 N for RECS100 contents of 1.5% and 3.0%, respectively.

**Fig 11:**
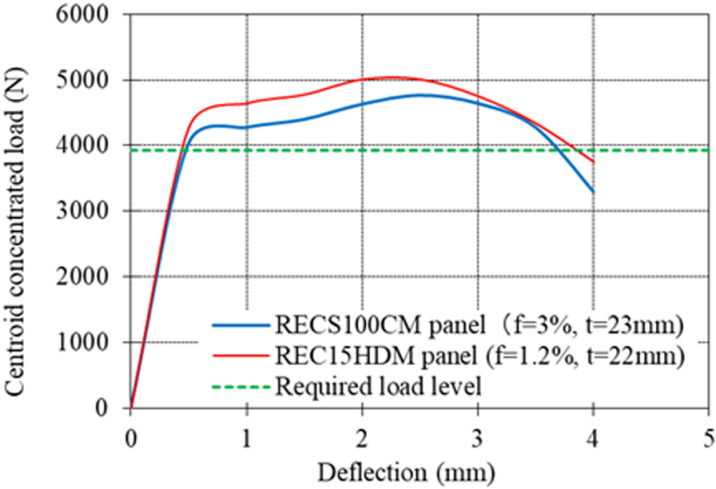
Load-deflection curve of REC15HDM and RECS100 CM panels.

Fig 11 shows the load-deflection curves of the REC15HDM and RECS100 CM panels. The REC15HDM panel had a thickness of 22 mm with 1.2% REC15 content, and the RECS100 CM panel had a thickness of 23 mm with 3.0% RECS100 content.

In the elastic range of the loading, the load-deflection curves of both types of panels were similar. However, the load capacity of the REC15HDM panel was found to be slightly higher than that of the RECS100 CM panel at the same level of deflection. Furthermore, the yield and maximum load capacities of the REC15HDM panels were higher than those of the RECS100 CM panels. Table 5 summarizes the necessary direct and indirect data for comparing the two panels.

**Table 5:** Comparison of the required mechanical properties of REC15HDM and RECS100 CM panels.

Items	REC15HDM	RECS100 CM
**Yield Load (N)**	4636	4270
**Maximum Load (N)**	5002	4758
**Deflection at maximum load (mm)**	2.02	2.53
**Stiffness at yield load (N/mm)**	8540	8052
**Stiffness at maximum load (N/mm)**	2001	1903

The yield and maximum load capacities of the REC15HDM panels were 8.57% and 5.13% higher, respectively, than those of the RECS100 CM panels. Similarly, stiffness at the yield level and maximum load level were higher by 6.06% and 5.15%, respectively. The deflection at maximum load of the REC15HDM panels was 20.2% less than that of the RECS100 CM panels. The deflections of both types of panels at the maximum load level were significantly less than the 5.0 mm limit. Both types of panels satisfied the maximum load and deflection requirements for OA flooring use. Overall, despite having less thickness and lower PVA fiber content, the REC15HDM panels performed better than the RECS100 CM panels.

According to JIS A 1408 [16], the panel should not exhibit any formation of cracks, and the residual deflection should be within 3.0 mm after the impact test. In this regard, a tested panel with large cracks and a residual deflection of more than 3.0 mm was termed “Worse.” And, a panel with minor cracks and a residual deflection of less than 3.0 mm was termed “Bad.” Furthermore, a panel with no cracks and a deflection of less than 3.0 mm was termed “Good.”

### Impact test results

**Fig 12:**
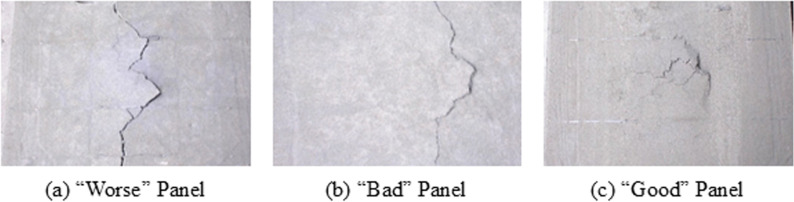
Classification of panels after the impact test.

Table 6 summarizes the results of the impact test. From the table, it is evident that only REC15HDM panels with a thickness of 22 mm and 1.2% REC15 fiber content, and RECS100 CM panels with a thickness of 23 mm and 3.0% RECS100 fiber content, met the requirements of JIS A 1408 [16]. This code specifies that panels must withstand a minimum compressive load of 3922.7 N and maintain a deflection within 5.0 mm. Additionally, it recommends that no cracks should form and residual deflection should not exceed 3.0 mm. In this study, only the panels that met the compressive load requirement also satisfied the impact resistance requirement. Images of samples classified as “Good,” “Bad,” and “Worse” are illustrated in Fig 12.

**Table 6:** Classification of panels after the impact test.

Panel types	Thickness (mm)	Fiber content (%)	Impact resistivity
**REC15HDM**	12	1.2	Worse
**REC15HDM**	15	1.2	Worse
**REC15HDM**	19	1.2	Worse
**REC15HDM**	20	1.2	Bad
**REC15HDM**	22	0.6	Worse
**REC15HDM**	22	0.8	Bad
**REC15HDM**	22	1.2	Good
**RECS100 CM**	23	1.5	Bad
**RECS100 CM**	23	3.0	Good

## Discussions

When fresh mortar is pressed to form thin panels with a thickness of 22 mm or 23 mm, the position of PVA fibers changes from their random orientation to an almost two-dimensional orientation along the length and breadth directions. When compressive or impact loads are applied to the upper surface of the panel, this two-dimensional fiber orientation enhances the load-resisting capacity and plays a major role in controlling the formation and propagation of cracks. McLean and Cui reported that fibers aligned in two dimensions have greater capacity to resist and distribute vertical loads effectively. This orientation optimizes the bridging capacity across cracks, thereby enhancing the overall mechanical performance and load-bearing capacity [39].

The enhancement in the mechanical properties of the panel, such as its resistance to compressive load and impact, is attributed to both the thinner size and the improved mechanical properties of the REC15 fiber compared to the RECS100 fiber. Due to the thinner size of the REC15 fiber, it significantly increases the number of fibers compared to RECS100, which enhances the panel’s crack resistance through more effective bridging. Additionally, the superior mechanical properties of REC15 fiber, as shown in Table 4, contribute to the enhanced performance of the REC15HDM panel, despite its lower fiber content compared to the RECS100 CM panel, which contains a higher fiber content.

While analyzing the total number of fibers in 1 m³ of panels, there were 41.45 × 10^9^ REC15 fibers in REC15HDM panels and 1.06 × 10^9^ RECS100 fibers in RECS100 CM panels. The number of REC15 fibers was found to be 39.1 times greater than the number of RECS100 fibers in 1 m³ of panels. This equates to 2,735,476 fibers in a single REC15HDM panel of 22 mm thickness and 183,028 fibers in a single RECS100 CM panel of 23 mm thickness. Despite the decrease in thickness by 1.0 mm and fiber content by 1.8%, the number of REC15 fibers in a single REC15HDM panel was still 14.9 times greater than the number of RECS100 fibers in a single RECS100 CM panel.

Let N denote the number of fibers uniformly distributed in a two-dimensional orientation throughout the length, breadth, and thickness of the panel. By knowing the total volume, $V_{p}$, of the panel, the spacing S of the uniformly distributed fibers can be calculated using the formula given in Equation (2).


$$S={\left(\frac{V_{p}}{N}\right)}^{\frac{1}{3}}$$ (2)

These fibers form grid structures throughout the length, breadth, and thickness of the panel. With the known numbers of fibers and volume of the panel, the spacing between REC15 fibers and RECS100 fibers was calculated to be 0.67 mm and 10.47 mm, respectively. This results in REC15 fibers being spaced 15.6 times closer together than RECS100 fibers. This indicates that the spacing between fibers decreases proportionally with the increase in the number of fibers. Specifically, the number of REC15 fibers in one panel was 14.9 times greater than that of RECS100 fibers. For instance, in the case of a linear crack formation of 100 mm length, bridging action is performed by approximately 150 numbers of REC15 fibers compared to only 10 numbers of RECS100 fibers.

Increasing the number of fibers enhances the resistance to crack formation and propagation in terms of both length and width before section failure [40]. Due to the 14.9-fold increase in the number of REC15 fibers compared to RECS100 fibers, the yield load and maximum load capacities of REC15HDM panels increased by 8.57% and 5.13%, respectively, in this study compared to RECS100 CM. Fig 10 shows that the load-deflection curve of the REC15HDM panel is slightly higher than that of the RECS100 CM panel, indicating that thinner fibers of the same length increase the toughness of fiber-reinforced cementitious composites [41]. Gyawali reported that thinner PVA fibers enhance the flexural strength of mortar composites more effectively than thicker PVA fibers [33]. The use of thinner PVA fibers also increases ductility, fracture energy, and cyclic loading capacities of mortar composites [41]. Furthermore, the failure of fiber-reinforced cementitious composites typically involves the formation of multiple cracks when thinner fibers are used [42].

### Cost analysis

Since the thickness and PVA fiber content of the REC15HDM panel were decreased from 23 mm to 22 mm and from 3.0% to 1.2% compared to the RECS100 CM panel, the cost of the REC15HDM panel has obviously decreased compared to the RECS100 CM panel. Given these dimensions, the volume of a single REC15 fiber and RECS100 fiber were calculated to be 24.127 × 10^-7^ liters and 9.4248 × 10^-7^ liters, respectively. With the same specific gravity of 1.3, the weight of a single REC15 fiber and RECS100 fiber were 3.1366 × 10^-8^ kg and 1.20 × 10^-6^ kg, respectively. This indicates that with 2.5 times decrease in diameter for the same length (12 mm), REC15 fiber is 39.1 times smaller and lighter than RECS100 fiber.

The length and width of both panels were similar, measuring 500 mm × 500 mm. The thickness of the REC15HDM panel and RECS100 CM panel were 22 mm and 23 mm, respectively. Therefore, the volumes of the REC15HDM panel and RECS100 CM panel were calculated to be 5.5 liters and 5.75 liters, respectively. This results in a 5.35% decrease in the volume of the REC15HDM panel compared to the RECS100 CM panel.

The volumes of REC15 and RECS100 fibers were calculated to be 0.066 liter and 0.1725 liter, respectively. Before calculating the weight of ingredients required for each type of panel, the mix proportions were adjusted to account for the discharged water during the pressing of the PVA mortar mix to form the panel. This adjustment resulted in the following unit contents: 276 kg/m³ water, 1150 kg/m³ cement, 921 kg/m³ sand, and 16.81 kg/m³ fiber for the REC15HDM mix, and 274 kg/m³ water, 1140 kg/m³ cement, 885 kg/m³ sand, and 41.66 kg/m³ fiber for the RECS100 CM mix. This gives weights for REC15 and RECS100 fibers of 0.086 kg and 0.240 kg, respectively. The weight of required fiber content in the REC15HDM panel decreased by 64.2% compared to the RECS100 CM panel.

The required amounts of cement and sand in one REC15HDM panel were 6.32 kg and 5.07 kg, respectively. In contrast, these values were 6.55 kg and 5.09 kg, respectively, for one RECS100 CM panel.

The costs of each kilogram of cement, sand, and PVA fiber were taken as $0.08, $0.019, and $5.0, respectively, based on average unit costs available in the market. This highlights that PVA fiber is the most expensive component in OA floor panel production. It is important to note that the cost of water is not included in this analysis due to its negligible expense compared to other ingredients.

Using the unit costs of each ingredient, the total material costs of the REC15HDM panel and RECS100 CM panel were calculated as US$ 0.92 and US$ 1.64, respectively. Assuming the costs of water, production, and manufacturing are the same for both panels, it can be inferred that overall costs could be reduced by 43.9% if REC15 fibers are used instead of RECS100 fibers for OA floor panel production. The details of the cost calculation per panel are summarized in Table 7.

**Table 7:** Summarized data of cost calculation.

Items	REC15HDM	RECS100 CM
**Sizes of panel (cm)**	50 × 50 × 2.2	50 × 50 × 2.3
**Volume of panel (liter)**	5.50	5.75
**Weight of fiber per panel (kg)**	0.086	0.240
**Weight of cement per panel (kg)**	6.32	6.55
**Weight of sand per panel (kg)**	5.07	5.09
**Unit cost of fiber (US$/kg)**	5.0	5.0
**Unit cost of cement (US$/kg)**	0.06	0.06
**Unit cost of sand (US$/kg)**	0.019	0.019
**Cost of fiber (US$)**	0.43	1.12
**Cost of cement (US$)**	0.39	0.41
**Cost of sand (US$)**	0.10	0.11
**Total cost (US$)**	0.92	1.64
**Decrease in cost (%)**	43.9	

**Fig 13:**
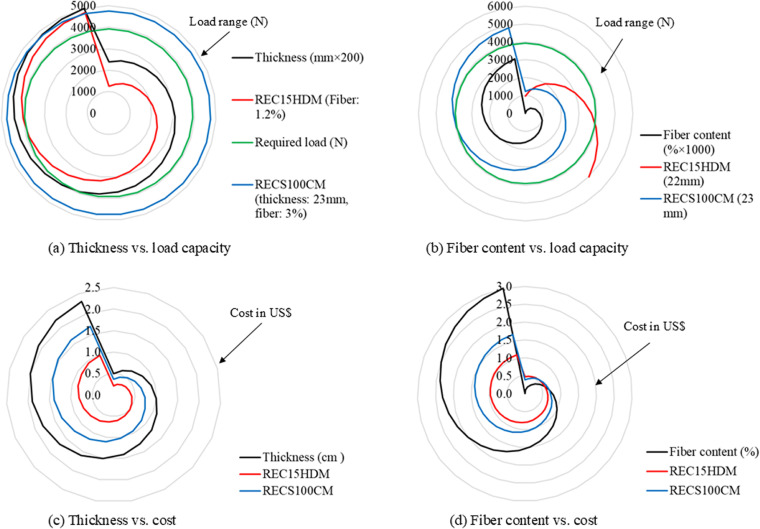
Compressive load resisting capacity and cost at varying thickness of panel and fiber content.

Furthermore, the load-resisting capacity and cost of the panels were analyzed across varying panel thicknesses and fiber contents. This analysis was performed using radar charts, following the method described in Luan et al.’s article [43]. A regression relationship was first established based on the test data, and the analysis of the compressive load-resisting capacity and cost of each panel was conducted, considering changes in thickness and fiber content. It should be noted that the compressive load-resisting capacity of RECS100 CM could not be analyzed, as its thickness was fixed at 23 mm, and no tests were conducted for varying thicknesses. Only the thickness of REC15HDM was varied for the analysis. However, both the thickness and fiber content of both panel types were varied for the cost analysis. Fig 13 presents the results, in radar charts, showing the compressive load-resisting capacity and cost for varying panel thickness and fiber content.

It is evident from Fig 13(a) that the compressive load-resisting capacity of the REC15HDM panel with 1.2% fiber content exceeded that of the RECS100 CM panel (thickness: 23 mm, fiber content: 3.0%) when the thickness of the REC15HDM panel was 22 mm. The load-resisting capacities of both panels were significantly higher than the required load of 3922.7 N. The load-resisting capacity of the REC15HDM panel reached 5027 N, even with a fiber content of just 1.2%, surpassing that of the 23 mm RECS100 CM panel (4875 N) with 3.0% fiber content (see Fig 13(b)).

In terms of cost, the REC15HDM panel was found to be significantly cheaper than the RECS100 CM panel across varying thicknesses (See Fig 13(c)). The cost difference became more pronounced as the thickness increased. For instance, the cost of the 22 mm REC15HDM panel with 1.2% fiber content was US$ 0.94, which is 43.0% less than that of the 23 mm RECS100 CM panel (US$ 1.65). This difference closely matched the actual cost variation (43.9%). A similar trend was observed when varying the fiber content (see Fig 13(d)). The cost of the 22 mm REC15HDM panel with 1.2% fiber content was 44.6% lower than that of the 23 mm RECS100 CM panel with 3% fiber content, again aligning with the actual cost calculation.

Summarizing all the above results, replacing thicker PVA fibers with thinner ones enhances the mechanical properties of the panel. It has been verified that achieving the required mechanical properties is possible not only by using less thinner fiber, with enhanced mechanical properties, but also by reducing the panel thickness. Additionally, replacing RECS100 fibers with REC15HDM fibers has significantly reduced the cost of the panel by almost half. The implementation of these findings supports the UN Sustainable Development Goals by reducing the carbon footprint associated with the production of cement and PVA fibers.

## Conclusions

The experimental work aimed to determine whether thicker PVA fibers (RECS100) could be effectively replaced by thinner PVA fibers (REC15) to achieve the required mechanical properties of OA floor panels. The high ductile mortar (HDM) method proved impractical for producing panels through pressing and water dehydration. The mortar’s proportions were adjusted slightly by adding 20 kg/m³ of water to maintain workability with REC15 fibers (REC15HDM), similar to RECS100 fibers (RECS100 CM), necessary for the pressing and dehydration process. The findings from the experimental investigations and analysis are summarized as follows:

- The REC15 fiber content in REC15HDM panels could be reduced to 1.2% from the 3% required in RECS100 CM with RECS100 fibers (a decrease of 60%).
- The thickness of REC15HDM panels could be reduced to 22 mm from the 23 mm required for RECS100 CM panels (a decrease of 4.35%).
- Both 22 mm thick REC15HDM panels and 23 mm RECS100 CM panels met the requirements for compressive load (3922.7 N) and deflection (≤ 5.0 mm). They also met the requirement for impact load (no cracks and residual deflection ≤ 3.0 mm).
- The yield and maximum load resisting capacity of REC15HDM panels increased by 8.57% and 5.13%, respectively, compared to RECS100 CM panels. Similarly, the stiffness at yield level and maximum load level of REC15HDM panels were 6.06% and 5.15% higher, respectively, than those of RECS100 CM panels. Additionally, the deflection of REC15HDM panels was 20.2% less than that of RECS100 CM panels.
- The number of REC15 fibers in a 22 mm thick REC15HDM panel was 14.8 times greater than the number of RECS100 fibers in a 23 mm thick RECS100 CM panel.
- The number of REC15 fibers involved in bridging a given length of crack was found to be 15.6 times greater than the number of RECS100 fibers.
- According to cost analysis, the cost of REC15HDM panels was reduced by 43.9% compared to RECS100 CM panels.

In conclusion, thinner PVA fibers are recommended to enhance the mechanical properties of OA floor panels, while significantly reducing production costs and promoting sustainability by lowering the carbon footprint associated with the production of cement and PVA fibers.

## Supporting information

S1 File XXXX. (ZIP)

S2 File XXXX. (ZIP)
