# Supplementary figures and images for: Effectiveness of thinner polyvinyl alcohol fibers on mechanical properties and cost effectiveness of office automation floor panels
Source: PLoS One. 2025 May 22;20(5):e0324126. doi: 10.1371/journal.pone.0324126 (PMC12097561; doi:10.1371/journal.pone.0324126)

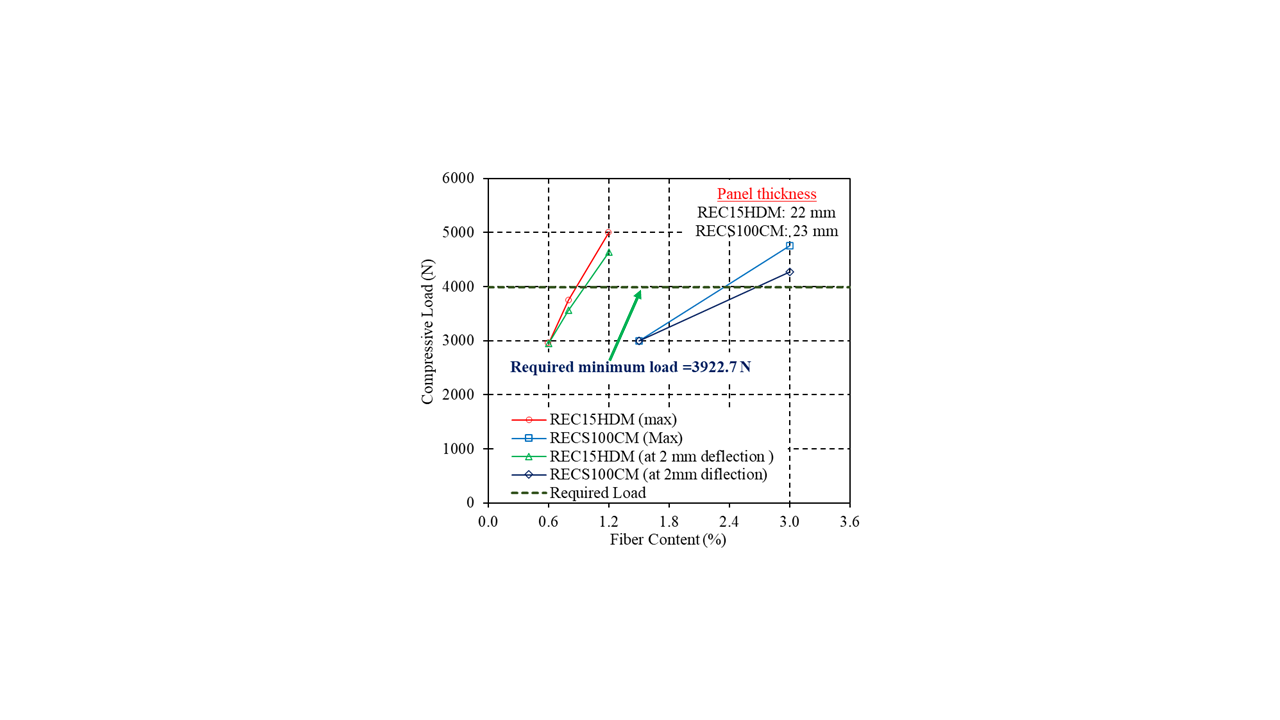

Supplement: S1 File — (ZIP) [file pone.0324126.s001.zip › Fig 10.tif]

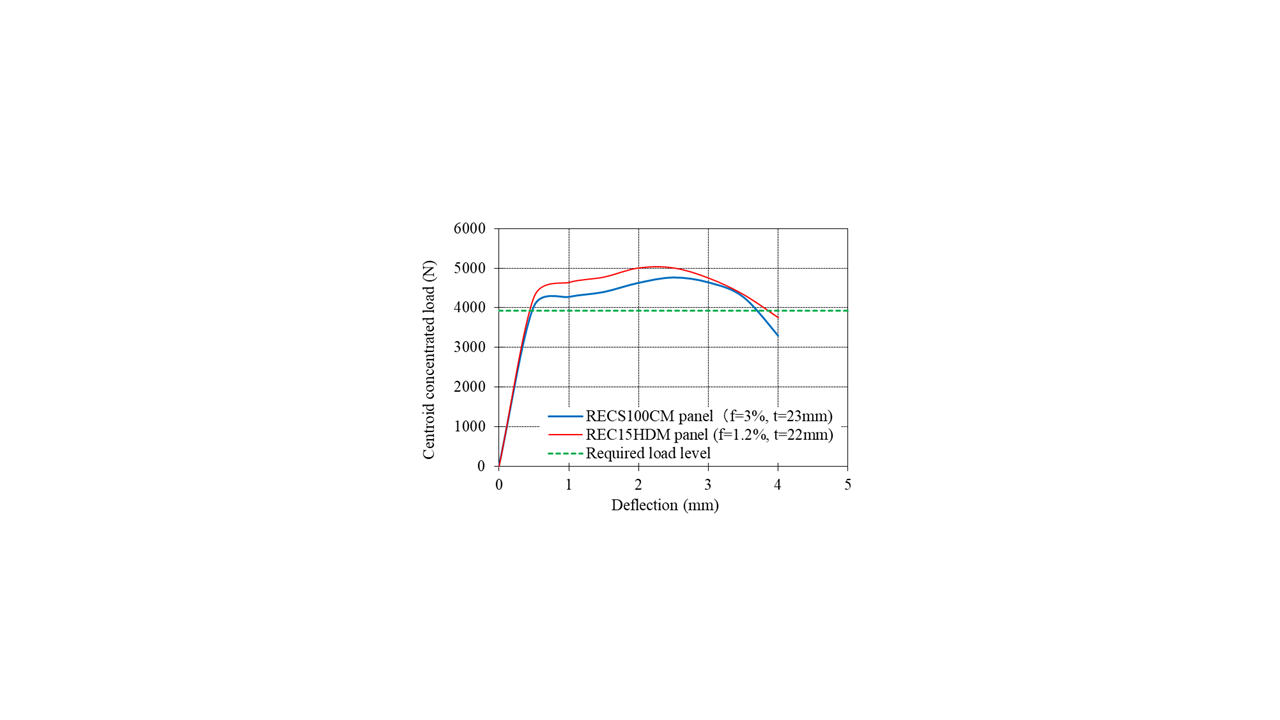

Supplement: S1 File — (ZIP) [file pone.0324126.s001.zip › Fig 11.tif]

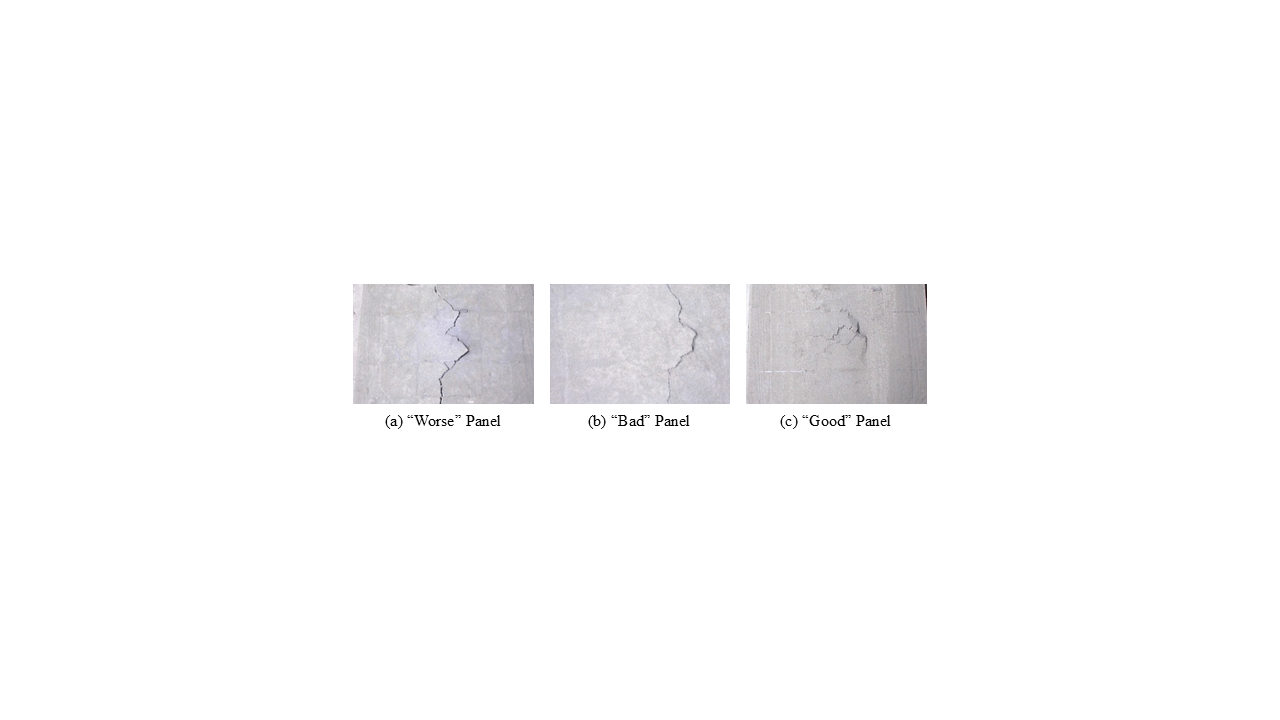

Supplement: S1 File — (ZIP) [file pone.0324126.s001.zip › Fig 12.tif]

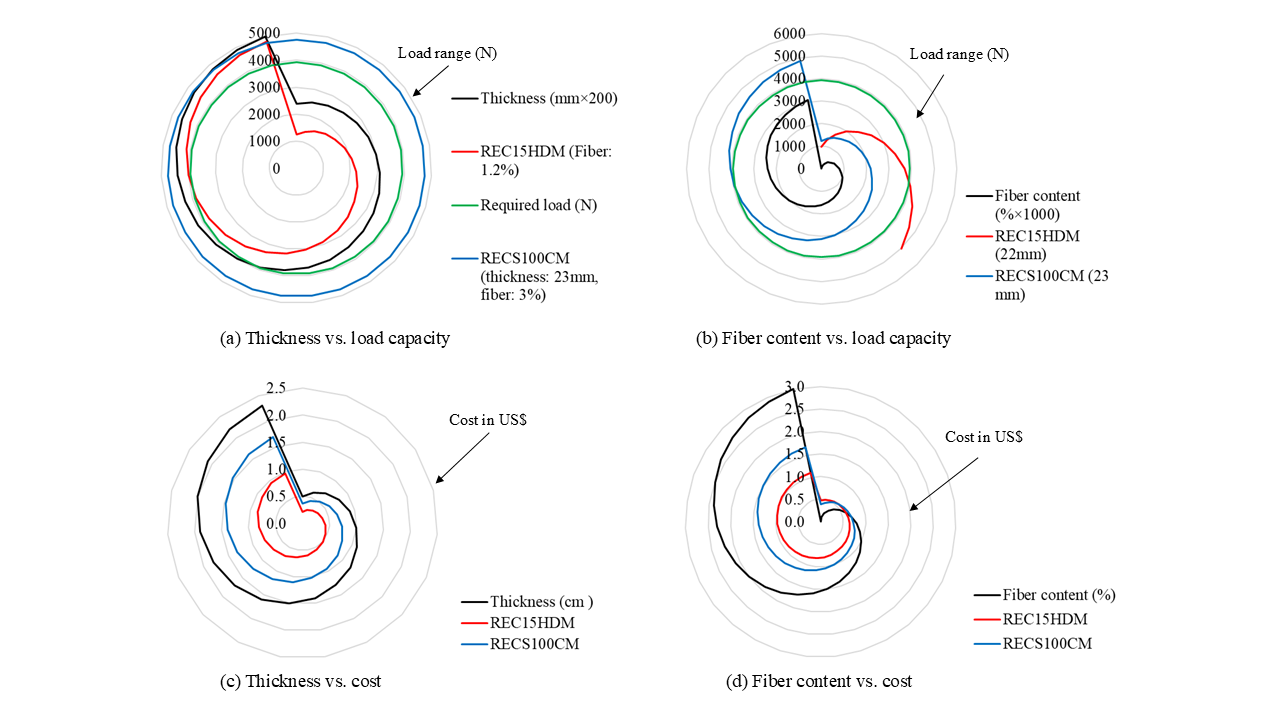

Supplement: S1 File — (ZIP) [file pone.0324126.s001.zip › Fig 13.tif]

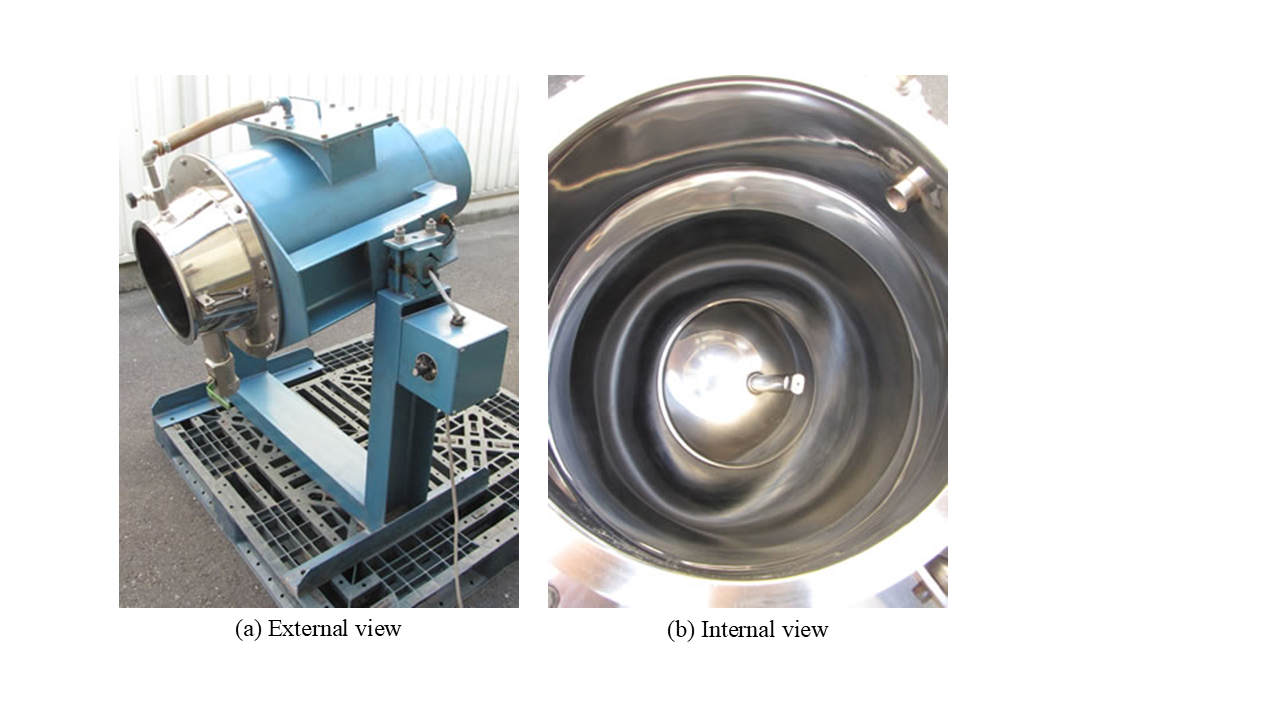

Supplement: S1 File — (ZIP) [file pone.0324126.s001.zip › Fig 1.tif]

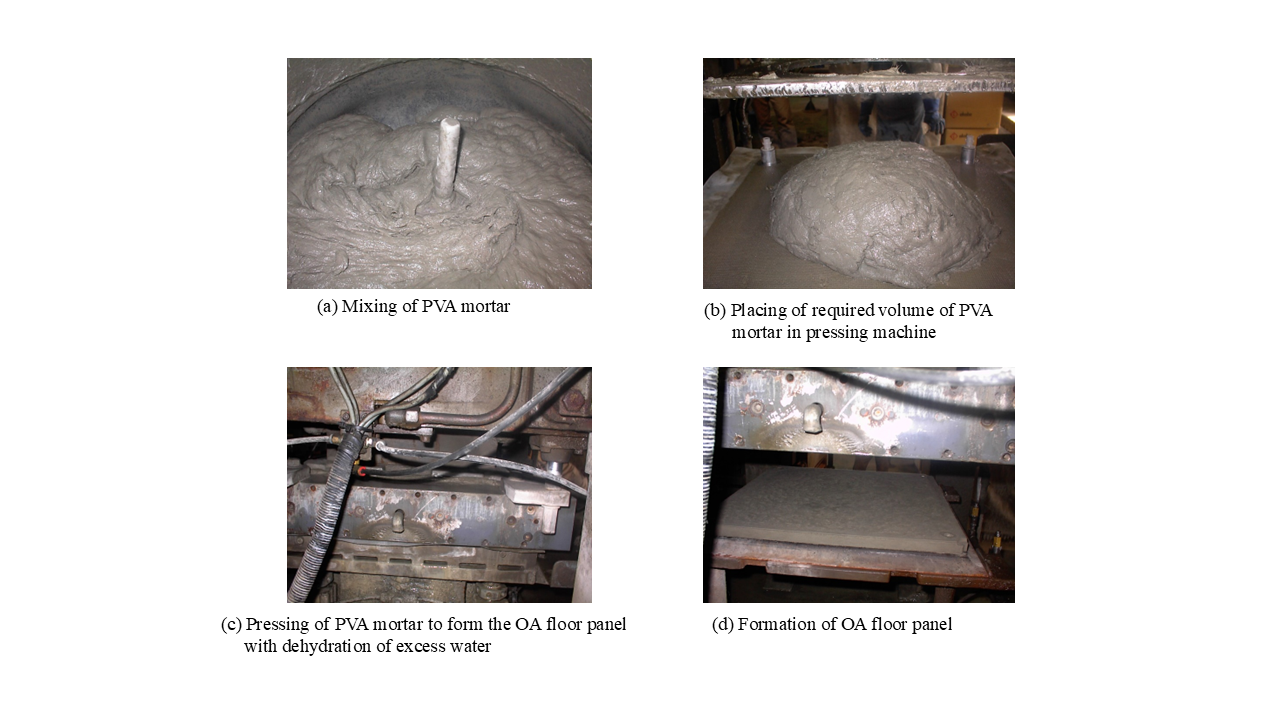

Supplement: S1 File — (ZIP) [file pone.0324126.s001.zip › Fig 2.tif]

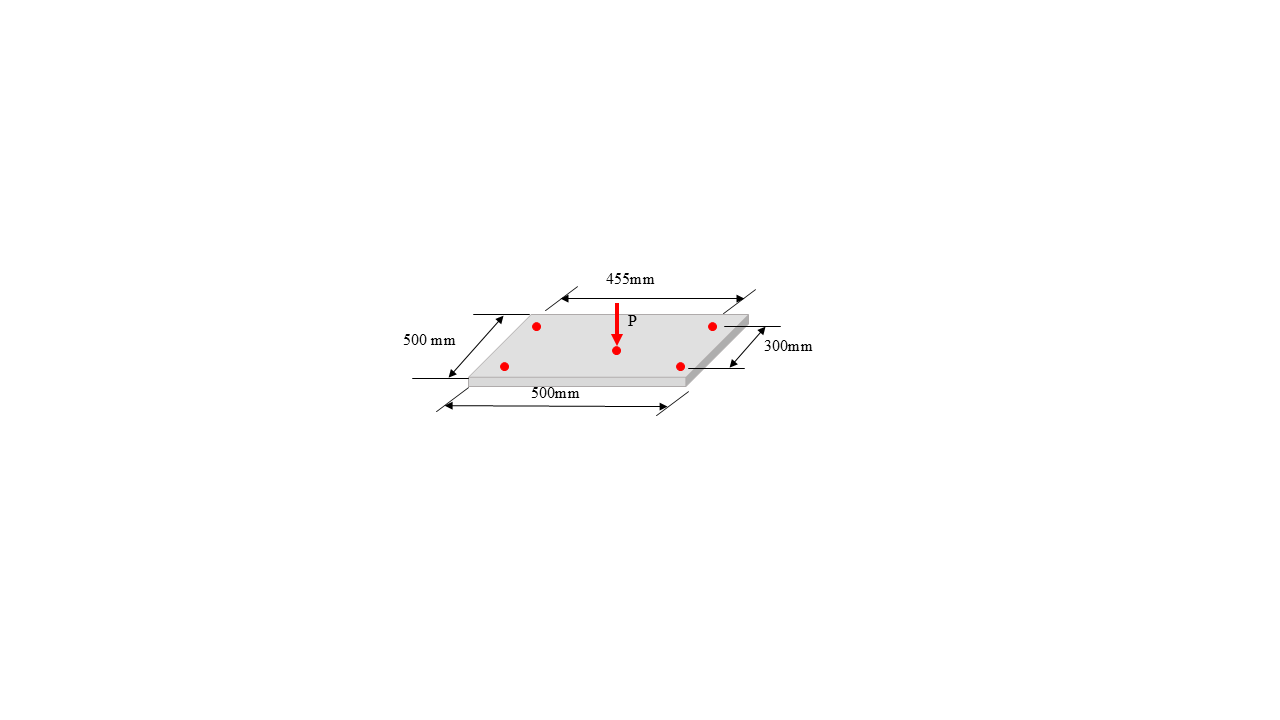

Supplement: S1 File — (ZIP) [file pone.0324126.s001.zip › Fig 3.tif]

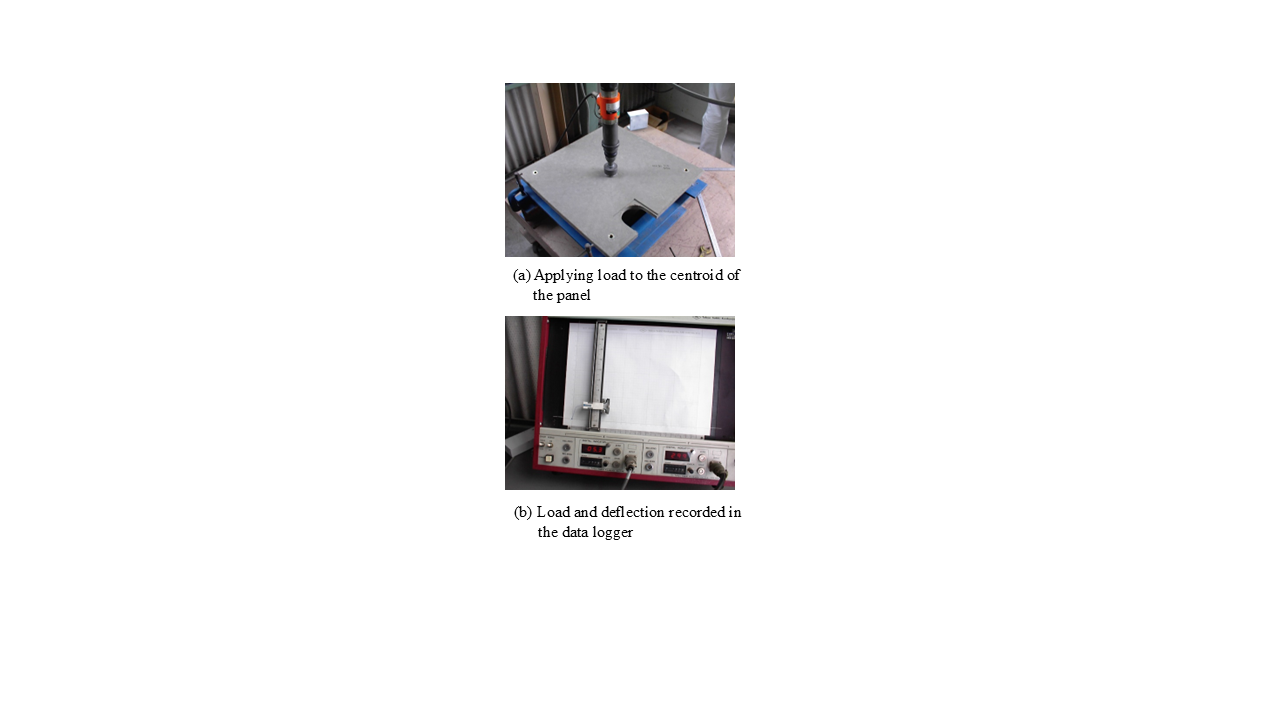

Supplement: S1 File — (ZIP) [file pone.0324126.s001.zip › Fig 4.tif]

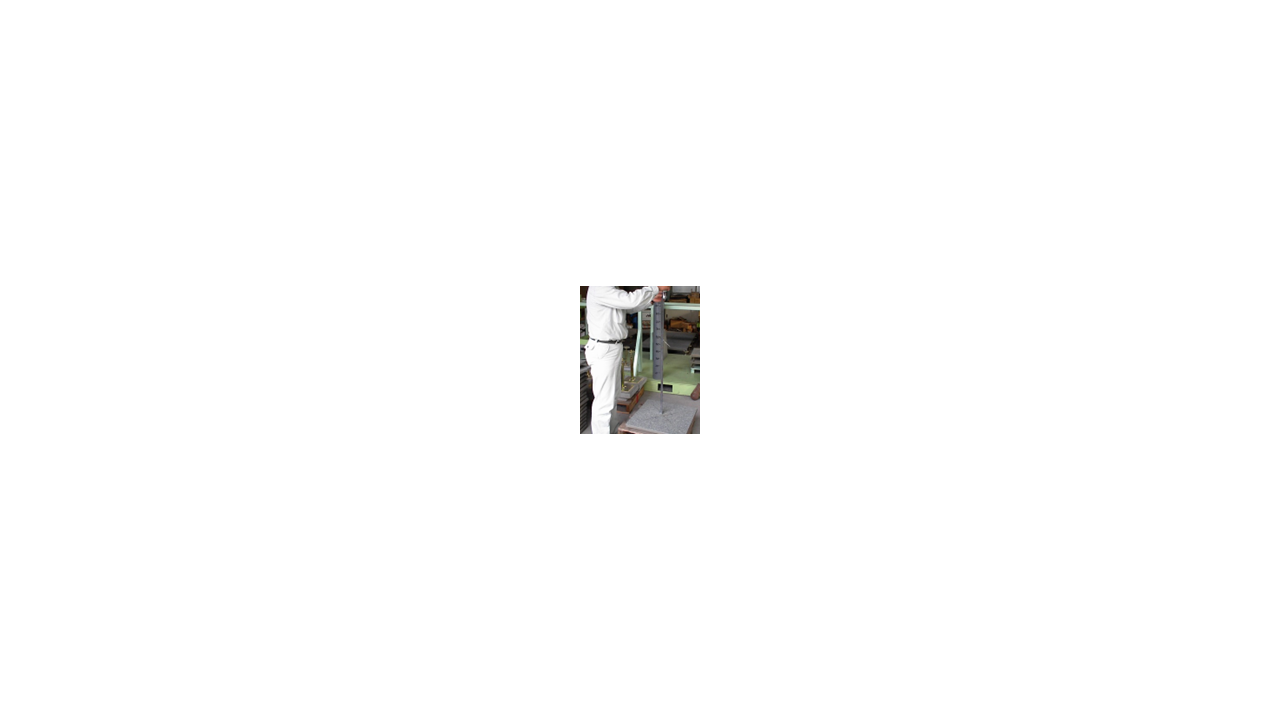

Supplement: S1 File — (ZIP) [file pone.0324126.s001.zip › Fig 5.tif]

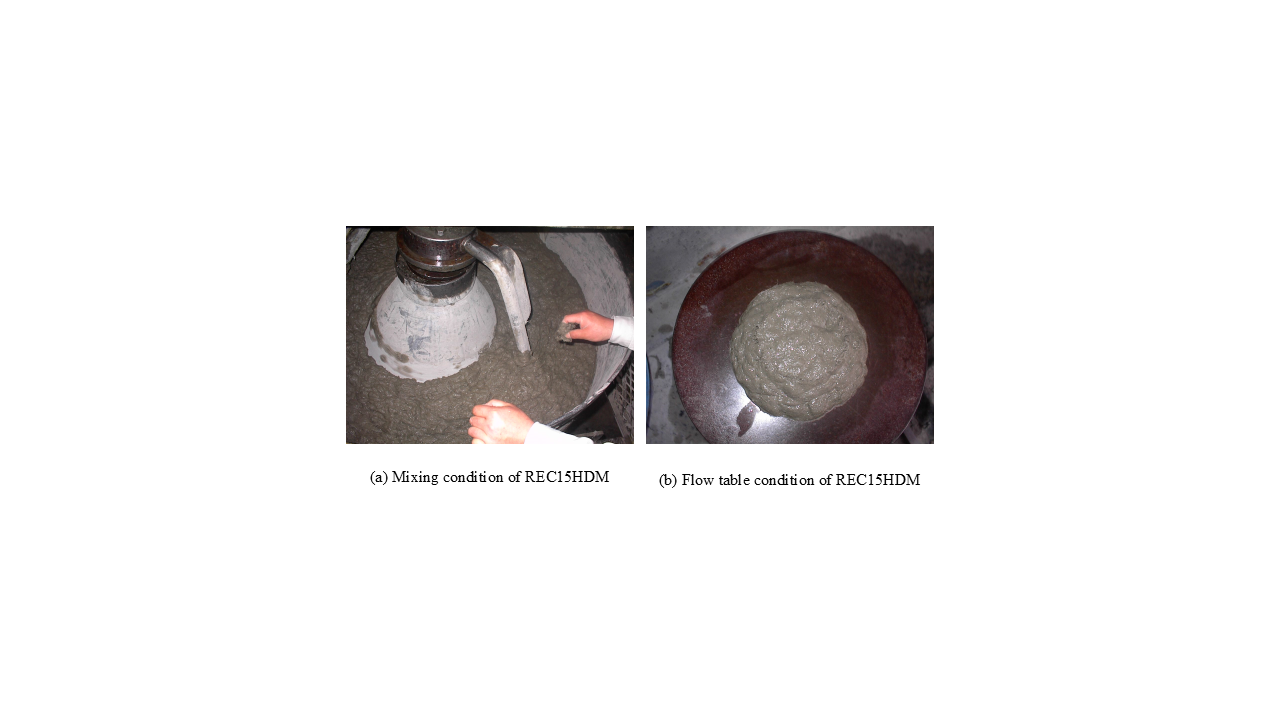

Supplement: S1 File — (ZIP) [file pone.0324126.s001.zip › Fig 6.tif]

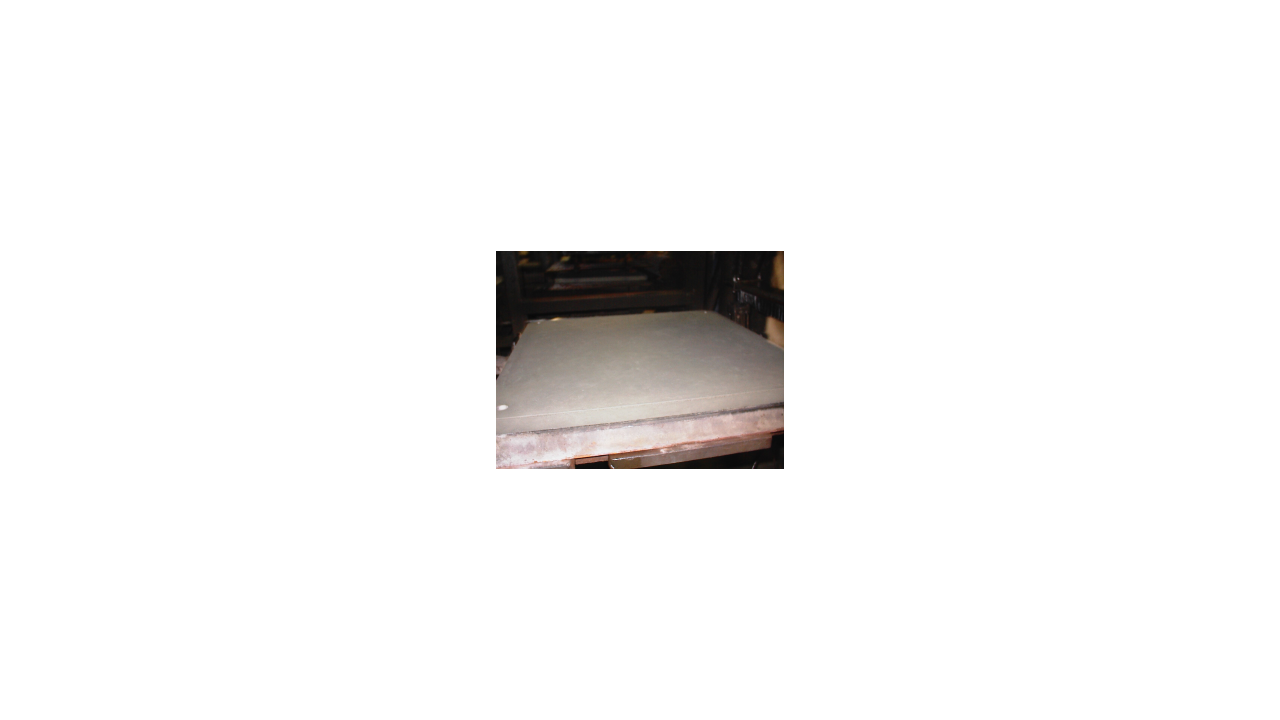

Supplement: S1 File — (ZIP) [file pone.0324126.s001.zip › Fig 7.tif]

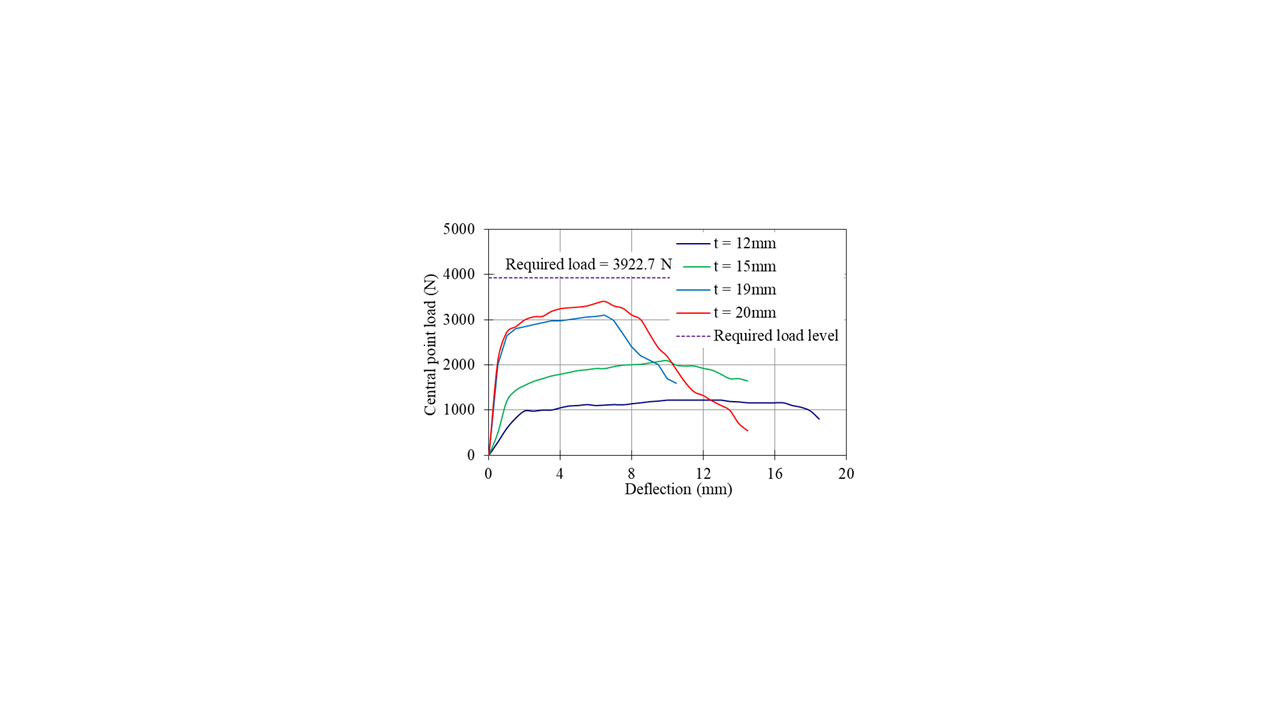

Supplement: S1 File — (ZIP) [file pone.0324126.s001.zip › Fig 8.tif]

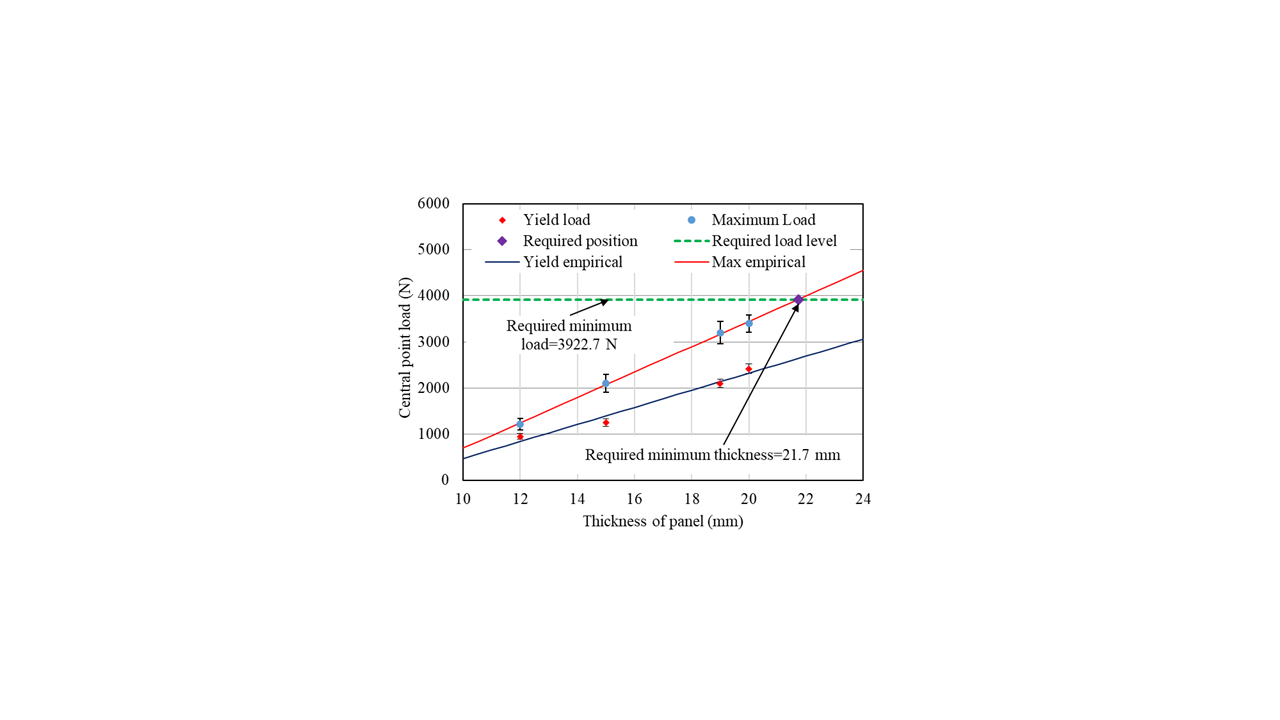

Supplement: S1 File — (ZIP) [file pone.0324126.s001.zip › Fig 9.tif]
